# Strengthening facility-based immunization service delivery in local government areas at high risk for polio in Northern Nigeria, 2014-2015

**DOI:** 10.11604/pamj.supp.2021.40.1.25865

**Published:** 2021-11-12

**Authors:** Belinda Vernyuy Uba, Ndadilnasiya Endie Waziri, Adekunle Akerele, Oladayo Biya, Oluwasegun Joel Adegoke, Saheed Gidado, Gideon Ugbenyo, Edwin Simple, Nnamdi Usifoh, Adamu Sule, Beza Kibret, Richard Franka, Eric Wiesen, Hashim Elmousaad, Chima Ohuabunwo, Lisa Esapa, Frank Mahoney, Omotayo Bolu, John Vertefeuille, Patrick Nguku

**Affiliations:** 1 National Stop Transmission of Polio, African Field Epidemiology Network, Abuja, Nigeria,; 2 Global Immunization Division, United States Centers for Disease Control and Prevention, Atlanta, Georgia,; 3 International Federation of Red Cross, Geneva, Switzerland

**Keywords:** Routine immunization, service delivery, polio, high risk

## Abstract

**Introduction:**

The National Stop Transmission of Polio (NSTOP) program was created in 2012 to support the Polio Eradication Initiative (PEI) in Local Government Areas (LGAs) at high risk for polio in Northern Nigeria. We assessed immunization service delivery prior to the commencement of NSTOP support in 2014 and after one year of implementation in 2015 to measure changes in the implementation of key facility-based Routine Immunization (RI) components.

**Methods:**

The pre- and post-assessment was conducted in selected health facilities (HFs) in 61 LGAs supported by NSTOP in 5 states. A standardized questionnaire was administered to the LGA and HF immunization staff by trained interviewers on key RI service delivery components.

**Results:**

At the LGA level, an increase was observed in key components including availability of updated Reach Every Ward (REW) micro-plans with identification of hard to reach settlements (65.6% baseline, 96.8% follow-up, PR = 1.5 (95% CI 3.4 - 69.8), vaccine forecasting (77.1% baseline, 93.5% follow-up, PR =1.2 (95% CI 1.8 - 13.8), and timely delivery of monthly immunization reports (73.8% baseline, 90.2% follow-up; PR =1.2 (95% CI 1.2 - 9.0). At the HF level, there was an increase in percentage of HFs with written supervisory feedback (44.5% baseline, 82.5% follow-up, PR = 1.8 (95% CI 4.7 - 7.3), written stock records (66.5% baseline, 87.9% follow-up, PR = 1.3 (95% CI 2.9 - 4.7) and updated immunization monitoring charts (76.3% baseline, 95.6% follow-up, PR = 1.3 (95% CI 4.6 - 9.9).

**Conclusion:**

We observed an improvement in key RI service delivery components following implementation of NSTOP program activities in supported LGAs.

## Introduction

Strengthening facility-based immunization service delivery in Nigeria has consistently required health systems strengthening and extension of its reach to underserved and hard-to-reach populations [1,2]. In 2012, as a component of the Global Emergency Action Plan, Global Polio Eradication Initiative (GPEI) partners were urged to ensure that vaccination coverage increased among underserved populations to eliminate the persistence of Wild Poliovirus (WPV) transmission. Limited facility-based service delivery was a backdrop to the surge in WPV cases and the persistence of transmission in Nigeria among the underserved populations during 2011-2016 [2,3].

In July 2012, the National Stop Transmission of Polio (NSTOP) program was established in Nigeria to support the implementation of the Global Emergency Action Plan in LGAs, which are responsible for immunization service delivery in health facilities (HF). The program is implemented by the National Primary Health Care Development Agency (NPHCDA) in collaboration with the United States Centers for Disease Control and Prevention (CDC), the Nigeria Field Epidemiology and Laboratory Training Program (NFELTP), and the African Field Epidemiology Network (AFENET). It is modeled after the international Stop Transmission of Polio program conducted by the CDC with the World Health Organization (WHO), and UNICEF. NSTOP local officers (NSLOs) were deployed to the LGAs in Northern Nigeria that were assessed as very high-risk for WPV transmission to strengthen HF-based immunization service delivery at LGA level [3-5] through i) capacity building for human resources delivering immunization services; ii) improvement in micro-planning, using the Reach Every Ward (REW) (Wards are subdistricts within LGAs) approach, which is aligned with the WHO´s Reach Every District (RED) strategy [5,6] to ensure more infants are reached; iii) improvement in coordination and planning among HF-based immunization service delivery stakeholders, and supportive supervision in health facilities providing immunization services [6,7]; iv) support for the implementation of supplemental immunization activities. The deployment occurred in phases, and the last (3rd) phase of officers were deployed in 2014 across 81 high risk LGAs in six states - Adamawa, Bauchi, Kano, Sokoto, Taraba and Borno. Upon commencement, officers went through modular trainings with post-training field assignments, focused on improving HF-based immunization service delivery.

To assess the impact of NSTOP support on HF-based immunization service delivery implementation in high-risk LGAs, a baseline assessment was conducted prior to the commencement of NSTOP support and a follow-up assessment was conducted one year later. The specific objectives of each assessment were to: 1) assess the availability of key immunization service delivery components at the LGA and HF levels; 2) compare the status of vaccination coverage indicators at baseline and follow-up; 3) identify opportunities for additional improvements in HF-based immunization service delivery.

## Methods

### Study area

**Figure 1:**
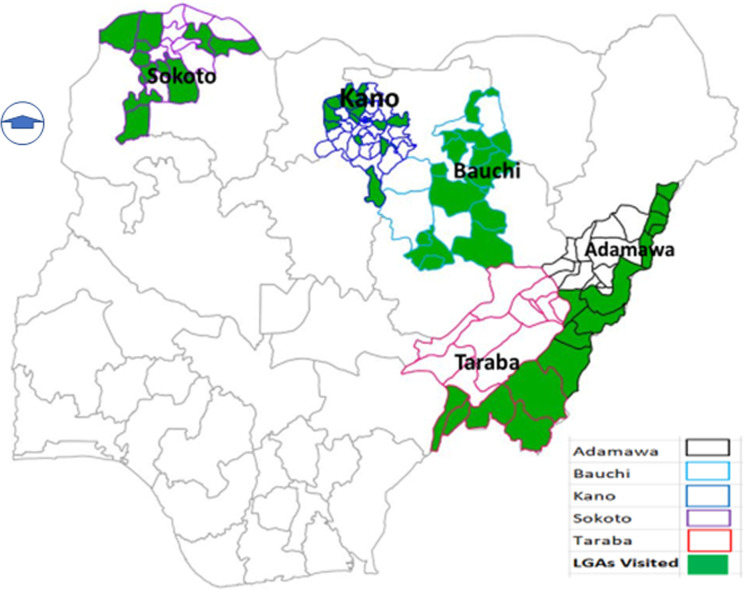
Map of Nigeria highlighting 61 local government areas supported by the national stop transmission of polio program in the states 2014-2015

We selected 61 phase 3 LGAs supported by NSTOP in 5 states (Adamawa, Bauchi, Kano, Sokoto and Taraba) for the assessment (Figure 1). The 20 phases 3 LGAs in Borno state were not included in the assessment because of security and access constraints.

### Study design and sampling

A cross-sectional study was conducted which composed of a baseline assessment in December 2014 and a follow-up assessment in December 2015. Each assessment took a month with field activities lasting for 2 weeks. All the wards in the LGA except those inaccessible due to security issues were assessed. There was a total of 794 wards across 61 LGAs, however only 723 were assessed in baseline and 715 in follow-up. Wards that were not assessed in the baseline were automatically excluded from follow-up assessment. Two HFs were randomly selected from each ward in all the accessible LGAs. In wards with only one HF, that HF was automatically selected and assessed. The selection of HF to be visited in the ward was done through balloting. All HFs in the ward were assigned numbers on pieces of papers and dropped in a box from which one HF was randomly picked by the independent evaluator and visited for the assessment. Same procedure as maintained in follow-up assessment and same LGAs were assessed in both baseline and follow-up.

### Participants

LGA respondents were the LGA Immunization Officer (LIO), cold chain officer, and health educator. HF respondents were the HF in-charge (or management coordinator) and the HF-based immunization service delivery focal person.

### Data collectors

Data were collected by independent interviewers who were graduates of the Nigerian field epidemiology and laboratory training program with a minimum qualification of Master of Public Health and who could speak the local dialect. They were trained for two days with a one-day field activity exercise for hands-on experience.

### Data collection

We used structured interviewer-administered questionnaires that were based on the Expanded Program on Immunization review tool obtained from the WHO website [8]. Using these questionnaires, we collected similar information at the LGA and HF levels about REW micro-plans, the availability of key RI service delivery components, including the availability of updated micro-plans and updated HF vaccination coverage monitoring charts; functionality of available Cold Chain Equipment (CCE) and maintenance of the updated temp monitoring charts; conducting planned immunization sessions in HFs; demand creation activities; and supportive supervision of HFs by the LGA staff including monthly LGA feedback at HFs.

### Data sources

Data gathered from verbal responses from the respondents was further verified by visualization of the RI data tools used at the facility before it was documented as available.

### Data analysis

Data were analyzed using Epi info™ 7 (2007) and Microsoft Excel™ (2010). Univariate and bivariate analyses were conducted using SPSS version 23 [9] and Microsoft Excel. We calculated the proportion of LGAs and HFs with RI indicators and determined proportion ratios (PR) by dividing the proportion with an indicator in the post-implementation assessment with the proportion in the pre-implementation assessment. We used PR to compare the change in the two proportions while Farrington and Manning´s Score was used to determine 95% Confidence Interval (CI). For LGA-level analysis, data from the same 61 LGAs were analyzed. For health-facility level analysis, due to the absence of a unique identifier for a health facility in the health facility database, we could not separate the health facilities that were included in both baseline and follow-up from those that were only included in either. The outcome indicators we analyzed at the LGA level included: i) the availability of updated REW micro-plans; ii) vaccine stock forecast availability with written stock records; iii) updated cold chain monitoring; iv) supportive supervision visits and availability of written supervisory feedback; v) updated HF vaccination coverage monitoring charts; vi) social mobilization activities conducted before the outreach sessions. At the HF level, the outcome indicators assessed included the availability of trained personnel providing immunization services, fixed and outreach sessions conducted, in addition to the indicators assessed at the LGA level.

### Ethical approval

Approval was obtained from the NPHCDA and the State Primary Health Care Board, and oral informed consent was obtained from each respondent.

## Results

Of the 794 wards across 61 LGAs, a total of 884 HFs in 723 wards were assessed at the baseline, and 861 HFs in 715 wards were assessed at follow-up (Table 1). At the LGA level, an increase was observed in the proportion of LGAs that had ward maps with identification of hard to reach areas (65.6% baseline, 96.8% follow-up, PR = 1.5 (95% CI 3.4 - 69.8)), updated micro plans (82.5% baseline, 94.2% follow-up, PR = 1.1 (95% CI 0.9 - 13.4)), written vaccine forecast availability (77.1% baseline, 93.5% follow-up, PR = 1.2 (95% CI 1.3 - 13.8)) and timely reports (73.8% baseline, 90.2% follow-up, PR = 1.2 (95% CI 1.2 - 9.0)) (Table 2).

**Table 1:** number of wards and health facilities (HFs) assessed in Local Government Areas (LGAs) supported by the National Stop Transmission of Polio Program, by State 2014-2015

State	Number of LGAs	Number of wards in the LGAs	Baseline 2014		Follow-up 2015	
			Number of Assessed Wards	Number of HFs	Number of Assessed Wards	Number of HFs
Adamawa	10	112	111	190	106	140
Bauchi	14	240	213	239	201	272
Kano	18	211	200	230	197	225
Sokoto	14	173	147	173	156	160
Taraba	5	58	52	52	55	70
**Grand Total**	**61**	**794**	**723**	**884**	**715**	**867**

**Table 2:** key Routine Immunization (RI) service delivery components in Local Government Areas supported by the National Stop Transmission of Polio Program, 2014-2015

Key RI components	Baseline (N=61) n (%)	Follow up (N=61) n (%)	Proportion Ratio (% Follow up/% at Baseline)	95 % Confidence Interval (CI)
**Planning and service delivery**				
Availability of Reaching Every Ward (REW) micro-plans	57 (93.5)	52 (85.3)	0.9	0.1 - 1.4
Updated REW plans	47 (82.5)	49 (94.2)	1.1	0.9 - 13.4
All components of REW captured in catchment area maps	49 (80.4)	57 (93.5)	1.2	1.1 - 11.5
Identified underserved communities in plans	40 (65.6)	59 (96.8)	1.5	3.4 - 69.8
Planned Fixed RI sessions conducted	32 (52.5)	45 (74.0)	1.4	1.2 - 5.5
Planned RI outreach sessions conducted	35 (59.0)	44 (72.0)	1.2	0.8 - 3.8
Median Fixed sessions conducted (Range)	29 (11-161)	25 (12-78)	-	-
Median Outreach sessions conducted (Range)	28 (10-125)	24 (11-73)	-	-
**Vaccine and stock management**				
Vaccine forecast availability	47 (77.1)	57 (93.5)	1.3	1.3 - 13.8
Availability of written vaccine supplies stock records	57 (93.5)	59 (96.8)	1.0	0.4 - 11.8
Inventory of vaccine Stock of antigens	17 (21.2)	26 (32.5)	1.6	0.9 - 4.1
**Cold chain monitoring:**				
Focal person for cold chain maintenance	59 (96.8)	59 (96.8)	1.0	0.1 - 7.3
Functional Fridges with updated temperature monitoring charts in LGA cold stores	61 (100.0)	61 (100.0)	1.0	0.00
**Supervision**				
Availability of supervisory work plan	53 (86.9)	61(100)	1.2	0 .00
Supervisory visits conducted with checklist	40 (65.6)	57 (93.5)	1.4	2.4 - 23.5
Median supervisory visits by LIO in a quarter	8 (0-58)	11 (0-90)		
Median supervisory visits by WFP in a quarter	4 (0-36)	3 (0-90)		
Availability of filled supervisory checklist	40 (65.6)	57 (93.5)	1.4	2.4 - 23.5
Availability of written feedback	25 (41)	50 (82)	2.0	2.9 - 15.0
**Monitoring**				
Updated RI monitoring charts	61 (100)	57 (93.5)	0.9	1.7 - 2.5
Timely Reports	45 (73.8)	55 (90.2)	1.2	1.2 - 9.0
**Communication**				
Availability of social mobilization plan	15 (24.6)	20 (32.8)	1.3	0.6 - 3.3

In a quarter of a year, a median of 29 (range 11-161) fixed sessions and 28 (range 10-125) outreach sessions were scheduled at baseline across all HFs, compared to 25 (range 12-78) fixed sessions and 24 (range 11-73) outreach sessions in the follow-up. The proportion of planned immunization sessions that were conducted increased from 52.5% at baseline to 74.0% at follow-up for fixed sessions; PR = 1.4 (95% CI 1.2 - 5.5) and from 59.0% at baseline to 72.0% at follow-up for outreach sessions; PR = 1.2 (95% CI 0.8 - 3.8). The proportion of conducted supportive supervision visits from the LGA to the HFs with use of checklist increased from 65.6% at baseline to 93.5% at follow-up; PR = 1.4 (95% CI 2.4 - 23.5). A median of 8 (range 0-58) supportive supervision visits were conducted by LIOs at baseline vs 11 (range 0-90) at follow-up, and 4 (range 0-36) conducted by officers at the ward level (Ward Focal Persons) in a quarter at baseline vs. 3 (range 0-90) at follow-up.

At HF level, an increase was observed in proportion of HFs with written feedback from supervisors (44.5% baseline, 82.5% follow-up, PR = 1.8 (95% CI 4.7 - 7.3)), availability of REW micro-plans (63.9% baseline, 86.6% follow-up, PR = 1.4 (95% CI 2.9 - 4.6)), updated vaccination coverage monitoring charts (76.0% baseline, 91.9% follow-up, PR = 1.2 (95% CI 2.6 - 4.7)), and HFs with updated temperature monitoring charts (17.9% baseline, 30.4% follow-up, 3PR = 1.7 (95% CI 1.5 - 2.6)). The proportion of planned, fixed, and outreach sessions conducted in HFs providing immunization services increased from 50.5% at baseline to 64.4% at follow-up; PR= 1.3 (95% CI 1.5 - 2.2) and 40.1% at baseline to 60.8 at follow-up; PR = 1.5 (95% CI 1.9 - 2.8) respectively, as well as increases in other key components of immunization service delivery at HF level (Table 3).

**Table 3:** key Routine Immunization (RI) service delivery components at the health facility (HF) level in Local Government Areas (LGAs) supported by the National Stop Transmission of Polio Program, 2014- 2015

Availability of key components at HF	Baseline (N=884)	Follow up (N=867)	Proportion Ratio (% Follow up/% at Baseline)	95 % Confidence Interval (CI)
	n (%)	n (%)		
**Human Resource**				
Trained personnel offering RI services	773 (87.5)	761 (87.8)	1.0	0.8 - 1.4
**Planning and service delivery**				
Availability of completed catchment area maps	633 (71.7)	741 (85.5)	1.2	1.8 - 3.0
Identified hard to reach/ underserved communities	468 (53.0)	608 (70.2)	1.3	1.7 - 2.5
Availability of REW micro plans	564 (63.9)	750 (86.6)	1.4	2.9 - 4.6
Updated REW micro plans*	505 (90.0)	637 (85.0)	0.9	0.4 - 0.9
Planned RI Fixed sessions conducted	443 (50.2)	558 (64.4)	1.3	1.5 - 2.2
Planned RI Outreach sessions conducted	354 (40.1)	527 (60.8)	1.5	1.9 - 2.8
**Vaccine and stock management**				
Focal person for cold chain maintenance	649 (73.5)	762 (87.9)	1.2	2.0 - 3.4
Vaccine forecast availability	166 (18.8)	424 (49.0)	2.6	3.3 - 5.1
Vaccine and supply stock records	587 (66.5)	762 (87.9)	1.3	2.9 - 4.7
Availability of functional fridges	563 (57.4)	658 (64.9)	1.1	1.5 - 2.2
Updated temperature monitoring chart[1]+	101 (17.9)	200 (30.4)	1.7	1.5 - 2.6
Vaccine collected by HF from LGA**	706 (79.9)	529 (61.1)	0.8	0.3 - 0.5
**Supervision and monitoring**				
Received feedback	393 (44.5)	715 (82.5)	1.8	4.7 - 7.3
Availability of RI monitoring chart	671 (76.0)	795 (91.7)	1.2	2.6 - 4.7
Updated RI charts#	512 (76.3)	760(95.6)	1.3	4.6 - 9.9
**Communication**				
Focal person for social mobilization	407 (46.0)	555 (64.0)	1.4	1.7 - 2.5
Annual plan for social mobilization	93 (10.5)	184 (21.2)	1.9	1.8 - 3.0
Displayed RI posters at HFs	62 (7.0)	20 (2.3)	0.3	0.2 - 0.5
Functional Village Development Committees	762 (86.2)	802 (92.6)	1.1	1.4 - 2.7
Conducted social mobilization activities before outreach session	754 (85.3)	797 (91.9)	1.1	1.4 - 2.7

* Updated REW micro-plans based on 564 and 750 HFs that had micro-plans in baseline and follow-up respectively + Updated temperature charts based on 563 and 658 HFs with fridges at baseline and follow up respectively # Updated RI charts based on 671 and 795 HFs that had monitoring charts at baseline and follow-up, respectively **The push policy where vaccines are taken to HFs rather them visiting LGAs is encouraged.

## Discussion

NSTOP support was able to improve the infrastructure necessary to support HF-based immunization service delivery and aid this aspect of polio eradication efforts. There was a significant improvement in some key components of immunization service delivery measured within a year after NSTOP initiated support to states. Key improvements were recorded at follow-up in planning and service delivery, vaccine and stock management, supervision, monitoring and communication for effective immunization service delivery when compared with the baseline. The improvement in the number of personnel who received immunization service delivery training is in line with the NSTOP´s model of regular mentorship and capacity building of immunization service delivery personnel [3] in line with one of the nine transformative investment of the Global Routine Immunization Strategies and Practices to achieving better immunization outcomes [1].

Effective micro-planning for HF-based immunization sessions, a key component of the REW strategy [7], is aimed at maximizing the limited available resources to ensure equitable delivery of immunization services and ensuring that all settlements are reached either through fixed or outreach sessions held at identified sites. These micro-plans are expected to be updated quarterly to ensure that missed and new settlements are captured and that there is equitable access for all children within the facility´s catchment area. The assessment identified an increase in the availability of updated REW micro-plans at both LGA and HF levels though a lot of activities involved in REW update were affected by the irregular release of funds from the state government for the development of these plans. However, most HFs recorded an increase in the number of immunization sessions conducted and a reduction in cancellation of sessions. This finding could be attributed to the technical support, mentorship, and improved supportive supervision provided for these facilities by NSLOs which was a key deliverable in the terms of reference. Additionally, the irregular release of funds for immunization services to HFs could have affected the expected improvement in the conduct of outreach sessions where transportation logistics have been identified as being critical. The reported insecurity challenges could have also attributed to the interruption of immunization services in HF and at outreach sites.

Reported stock-outs of some vaccines in HFs could be attributed to the pull system of vaccine delivery, which requires a visit from HF staff to the LGA, and a visit from LGA staff to the State, to collect vaccines. The pull system is often used in health facilities that do not have their own CCE, often collecting vaccines from the LGA on the day of the immunization session. A transportation or other logistical problems on the day of the immunization session could result in stock-out of vaccines [10]. Cold chain monitoring and maintenance improved among facilities that had CCE; however, the availability of CCE was not uniform across all HFs.

There was improvement in monitoring of data for action and communication for HF-based immunization service delivery. The availability of vaccination coverage monitoring chart that captures key immunization service delivery indicators regularly updated and routinely monitored in line with the immunization accountability framework indicators in the strategic plan [11] are critical for data-driven decision-making. NSLOs routinely supervise health facilities and mentor them on the use of updated vaccination coverage monitoring charts and tracking of the indicators on the charts to identify facilities not meeting the set targets which are then prioritized for follow-up supportive supervision.

Improvements were noted in the availability of supervisory plans, the proportion of the supervisory visits conducted among those planned, the availability of completed supervisory checklists and written supervisor feedbacks that identified follow-up actions. Although partner organization staff conduct most visits, LGA teams are usually paired with partners to conduct supportive supervisory visits to HFs to identify best practices and prioritize those HFs with challenges for follow-up actions [6].

The availability of monthly and annual reports also improved in the follow-up assessment from baseline, including feedback from the state and LGA to HFs. This can possibly be attributed to the mentorship provided at all levels, as well as the increased capacity of personnel on writing reports [12,13].

The availability of social mobilization and communication plans at both baseline and follow-up including the involvement and functionality of Ward Development Committees (WDCs)/Village Development Committees (VDCs) in immunization services at some facilities can be attributed to technical support and frequent community engagement activities conduct during supervision. This was identified as a critical area that needed support to raise community awareness, create demand, and clear misconceptions about immunization. NSTOP did not provide funding of WDCs/VDCs but technically facilitated and supported their meetings.

### Limitations

This study had some limitations; some HFs included in the baseline assessment within the same LGA were not the same HFs included in the follow-up assessment, and different numbers of HFs were assessed at each point in time. However, we believe that findings can be generalized to what is applicable in all HFs in the LGAs. Also, security challenges did not allow for a full assessment of all HFs due to inaccessibility in some areas. Lastly, in the before and after design, we did not include any non-intervention areas that would have allowed us to assess any effect of secular trends on program performance.

## Conclusion

This assessment showed an improvement in some of the key components of HF-based service delivery, after a one year of NSTOP support to the states. Key areas that provided opportunities for additional support to strengthen HF-based service delivery, especially in the measles elimination efforts and the control of other VPDs were also identified. These included tailored support on vaccine management and cold chain logistics, communication and social mobilization for demand generation for childhood immunization, optimization of the quality of supportive supervision for immunization services, regular capacity building for health workers at all levels, as well as support for the conduct of outreach sessions to ensure that HF-based services reach the unreached communities. Therefore, we recommend that LGAs (a) develop vaccine delivery mechanisms that will ensure a regular and adequate supply of vaccines and other logistics needed to conduct sessions, (b) strengthen social mobilization activities to forestall interruption of HF-based services, and (c) train personnel on preventive maintenance of available CCE. We also recommend that communication and demand generation activities to increase uptake of immunization should be prioritized and adequately supported by the government at all levels (national, state and LGA) to ensure community mobilization to fully immunize children and community ownership of immunization services through WDCs/VDCs as a component of primary health care.

### What is known about this topic

- It is well known in the immunization landscape that strengthening routine immunization using available structures and resources in the system is a key pathway to improving on primary health care delivery;
- Support to vertical programs like the polio eradication program can only be more effective and sustainable in achieving its goals when RI is strengthened, and services are integrated at the service delivery points in the primary health care centers.

### What this study adds

- This study was able to showcase how polio resources can effectively be used to support and strengthen RI and how to effectively integrate and institutionalize activities to ensure sustainability in the system;
- The study was able to showcase best practices in the system that were shared for institutionalization and gaps in the RI system were also identified and strengthened while areas of governments intervention were recommended and followed up for prompt actions.
